# Neuroinflammation in Alzheimer's disease: the role of the P2X7 receptor (P2X7R)

**DOI:** 10.1002/alz70855_102906

**Published:** 2025-12-24

**Authors:** Simonetta Falzoni, Selene Schio, Mario Tarantini, Anna Lisa Giuliani

**Affiliations:** ^1^ University of Ferrara, FERRARA, Ferrara, Italy

## Abstract

**Background:**

Growing evidence underscores the central role of neuroinflammation in Alzheimer's Disease (AD). In inflamed brain accumulation of pro‐inflammatory molecules promote the activation of resident immune and accessory cells, i.e. microglia and astroglia, which become dysfunctional, injure neurons and promote the accumulation of toxic factors, such as the amyloid‐β peptides. A key player in inflammation is extracellular ATP (eATP), a pro‐inflammatory molecule that accumulates to high concentrations at sites of inflammation. A main receptor for eATP is the purinergic P2X7 receptor (P2X7R) expressed by different cell types, especially of the immune system. P2X7R activation determines the recruitment of the NLRP3 inflammasome which provokes the maturation of pro‐IL‐1 beta to mature IL‐1 beta.

**Method:**

First, we measured, by the pmeLUC luminescent probe, the eATP level in the pericellular space of N13 murine microglial cells, expressing either high (N13 WT) or low (N13 R) levels of the P2X7R, stimulated with conditioned media from CHO 7PA2 cells genetically modified to produce beta‐amyloid peptides. We also evaluated the expression of P2X7R and NLRP3 and the release of IL‐1 beta in N13 WT and N13 R stimulated cells. Further, we studied the mRNA levels of P2X7R, NLRP3 and IL‐1 beta in primary microglial cells submitted to the same treatments.

**Result:**

We found that the treatment of N13 WT murine microglial cells with conditioned media from CHO 7PA2 cells induces the following effects respect to controls: 1) significantly augmented eATP levels in the pericellular space; 2) increased P2X7R and NLRP3 protein expression; 3) increment of IL‐1 beta release. The same treatment of primary microglial cells increases the P2X7R, NLRP3 and IL‐1 beta mRNA levels.

**Conclusion:**

N13 WT murine microglial cells treated with conditioned media from CHO 7PA2 cells containing beta‐amyloid peptides showed pro inflammatory features witnessed by increased eATP release, and augmented expression of the pro‐inflammatory molecules P2X7R, NLRP3 and IL‐1 beta. These features were significantly lower in N13 R cells, expressing low levels of the P2X7R. These results were substantially confirmed using primary microglial cells. Our results support the role of P2X7R in neuroinflammation and in AD, and pave the way to new possible therapeutic treatments for AD.

